# Human Induced Pluripotent Stem Cells Differentiation into Oligodendrocyte Progenitors and Transplantation in a Rat Model of Optic Chiasm Demyelination

**DOI:** 10.1371/journal.pone.0027925

**Published:** 2011-11-18

**Authors:** Alireza Pouya, Leila Satarian, Sahar Kiani, Mohammad Javan, Hossein Baharvand

**Affiliations:** 1 Department of Stem Cells and Developmental Biology, Cell Science Research Center, Royan Institute for Stem Cell Biology and Technology, ACECR, Tehran, Iran; 2 Department of Physiology, Faculty of Medical Sciences, Tarbiat Modares University, Tehran, Iran; 3 Department of Developmental Biology, University of Science and Culture, ACECR, Tehran, Iran; National Institute on Aging Intramural Research Program, United States of America

## Abstract

**Background:**

This study aims to differentiate human induced pluripotent stem cells (hiPSCs) into oligodendrocyte precursors and assess their recovery potential in a demyelinated optic chiasm model in rats.

**Methodology/Principal Findings:**

We generated a cell population of oligodendrocyte progenitors from hiPSCs by using embryoid body formation in a defined medium supplemented with a combination of factors, positive selection and mechanical enrichment. Real-time polymerase chain reaction and immunofluorescence analyses showed that stage-specific markers, Olig2, Sox10, NG2, PDGFRα, O4, A2B5, GalC, and MBP were expressed following the differentiation procedure, and enrichment of the oligodendrocyte lineage. These results are comparable with the expression of stage-specific markers in human embryonic stem cell-derived oligodendrocyte lineage cells. Transplantation of hiPSC-derived oligodendrocyte progenitors into the lysolecithin-induced demyelinated optic chiasm of the rat model resulted in recovery from symptoms, and integration and differentiation into oligodendrocytes were detected by immunohistofluorescence staining against PLP and MBP, and measurements of the visual evoked potentials.

**Conclusions/Significance:**

These results showed that oligodendrocyte progenitors generated efficiently from hiPSCs can be used in future biomedical studies once safety issues have been overcome.

## Introduction

Demyelinating diseases such as multiple sclerosis (MS) are characterized by damage to the myelin sheath surrounding neurons, causing impaired nerve impulses that lead to a constellation of neurological symptoms. Recent research on cell transplantation has yielded new insights into the novel possibilities of using stem cell-derived oligodendrocytes in graft-based remyelination therapy to restore action potential conduction. However, to date, an efficient and reliable cell source has not been introduced (for review, see [1]. The recent groundbreaking developments regarding induced pluripotent stem cells (iPSCs) generated from easily accessible somatic cells [2] appear to offer a nearly inexhaustible source of transplantable, autologous neural stem cells (for review, see [3], [4]. Many studies have demonstrated that mouse and human iPSCs are highly morphologically, molecularly and phenotypically similar to their respective embryo-derived embryonic stem cell (ESC) counterparts [5], [6]. The use of iPSCs also circumvents the ethical issue related to using ES cells and producing human disease models *in vitro* (for review, see [7].

Several studies have reported the functional maturation and neural and oligodendrocyte differentiation of ESCs *in vitro* as well as therapeutic use of ESCs in animal models of demyelinating diseases and remyelination after spinal cord injury [8]–[24]. These studies have suggested that ESC-derived oligodendrocyte progenitors (OPs) can potentially promote regeneration and reduce secondary degeneration by protecting and restoring axons.

To date, there have been two reports of differentiation of OPs from mouse iPSCs [25], [26]. However, there is no report of differentiation of OPs from human iPSCs (hiPSCs) and their transplantation into a demyelinating animal model. Based on the similarity of hiPSCs and human ESCs (hESCs), we hypothesized that generation and maturation of OPs from hiPSCs *in vitro* could be achieved with the same protocol used with hESCs. Therefore, we applied a previously described protocol for differentiating hESCs into oligodendrocyte lineage cells [27] to hiPSCs. Additionally, following transplantation, we investigated the capacity of the hiPSC-derived OPs to improve demyelinated optic chiasm in a rat model, which had been induced by lysolecithin.

## Results

### Differentiation and characterization of hiPSCs and hESCs into OPs and oligodendrocytes

**Figure 1:**
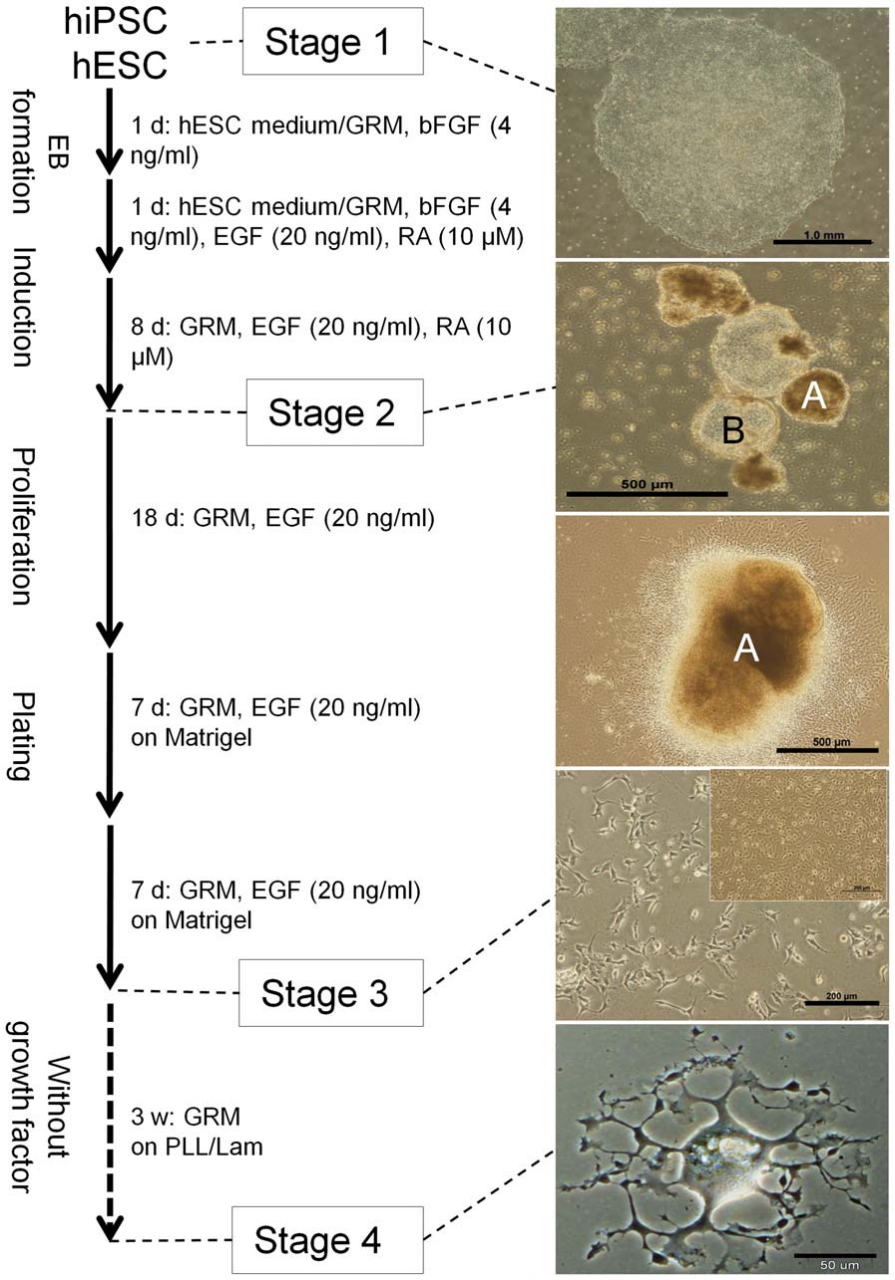
Differentiation protocol and cellular morphologies during oligodendroglial-lineage cell differentiation. hiPSCs (hiPSC1 and hiPSC8 lines) and hESCs (Royan H6 line) were differentiated to oligodendrocyte lineage cells during induction of neural-lineage cells with the preferential selection of oligodendroglial-lineage cells using media components. Morphologies of cells at the related stages are depicted. Two types of spheres were observed in stage 2: yellow spheres (A), which were plated on Matrigel for the subsequent stages, and transplant spheres (B), which were removed from cultures. The representative pictures showed hiPSC1 differentiation.

The differentiation procedure and the morphology of cells at different stages are illustrated in Figure 1. Selective oligodendrocyte differentiation from undifferentiated feeder-free hiPSCs (stage 1) was performed by forming EB and culturing the cells in a GRM/hESC medium containing 4 ng/ml bFGF at a 1∶1 ratio. The EBs were then exposed to GRM in the presence of RA and EGF for nine days (stage 2). This exposure led to the appearance of a transparent sphere of cells (Figure 1, B type) and yellow spheres of cells (Figure 1, A type) that contained oligodendrocyte-lineage cells. With the removal of RA and continuing the suspension cultures in GRM supplemented with EGF for 18 days, the size of the yellow spheres increased. At day 28, yellow-type free-floating spheres were plated on Matrigel. In the Matrigel cultures, cells migrated progressively outward from the spheres and were maintained and proliferated in the same medium. Cells were passaged (1∶2) twice every seven days (day 42, stage 3). At this step, cells displayed immature oligodendrocyte bipolar and multipolar morphologies, which were similar to those of OPs. To evaluate the oligodendrocyte maturation potential of these cells, induction was used by removing EGF from the medium, and cells were replated at a low density (10,000 cells/cm^2^) on a PLL/lam substrate for three weeks. After this treatment, plated cells adopted an oligodendroglial morphology characterized by multiple branches (stage 4). The same morphological results were observed for hESCs.

**Figure 2:**
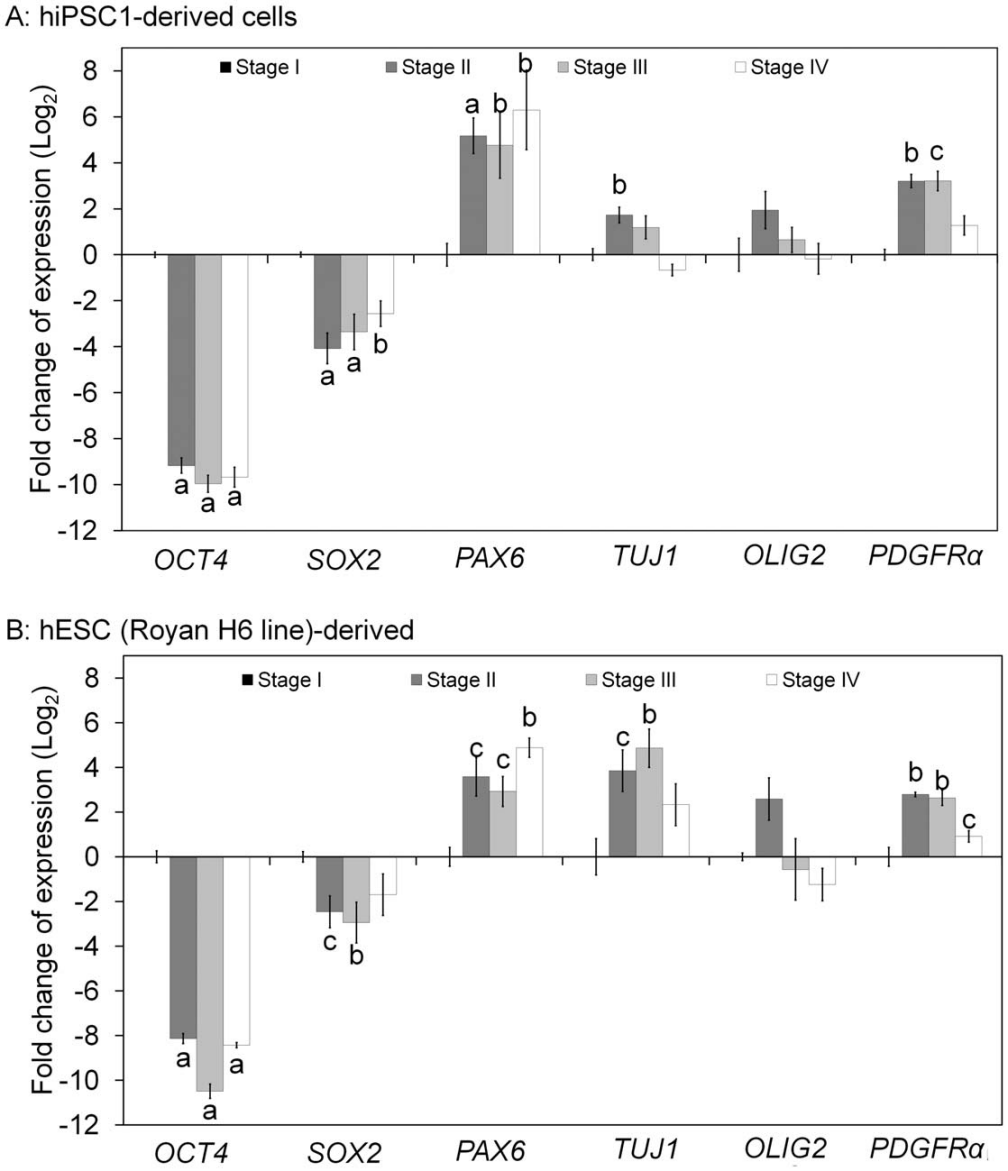
Relative gene expression using real-time PCR. Fold changes of expression were reported as log2. For real-time PCR, sampling was performed at four different stages during the differentiation protocol. Several primers were used for the pluripotent genes (Oct4 and Sox2), the neural lineage genes (Pax6 and Tuj1), and the oligodendrocyte lineage genes (Olig2 and PDGFRα). Expression of the pluripotent-specific genes decreased during the differentiation of hiPSC1(A) and hESC (Royan H6 line) (B) lines into oligodendrocytes when compared to stage 1. Expression levels of Pax6, Tuj1, and PDGFRα increased, but Tuj1 and PDGFRα expression decreased in stage 4. Gene expression levels at different hiPSCs and hESC-oligodendrocyte lineage cell stages relative to the levels in stage 1 approximate the gene expression levels of derived cells. c: p<0.05, b: p<0.01, a: p<0.001 vs. hiPSC1 or hESC stage; Error bar: SEM.

Cells were examined by qRT-PCR at different points of differentiation for expression of markers typical of oligodendrocyte development in hiPSCs and hESCs (Figure 2). *OCT4* and *SOX2*, markers of pluripotency, were down-regulated following induction. Additionally, we could not detect OCT4-positive cells by immunofluorescence staining at stages 3 and 4 in the hiPSC-derived OPs. Expression of the neural markers *PAX6* and *TUJ1* was up-regulated, and the expression of *TUJ1* decreased at stage 4 following additional maturation. The expression levels of *PDGFRα* and *OLIG2* (OPs markers) were up-regulated during OP induction (stages 2 and 3) and down-regulated after more differentiation (stage 4).

**Figure 3:**
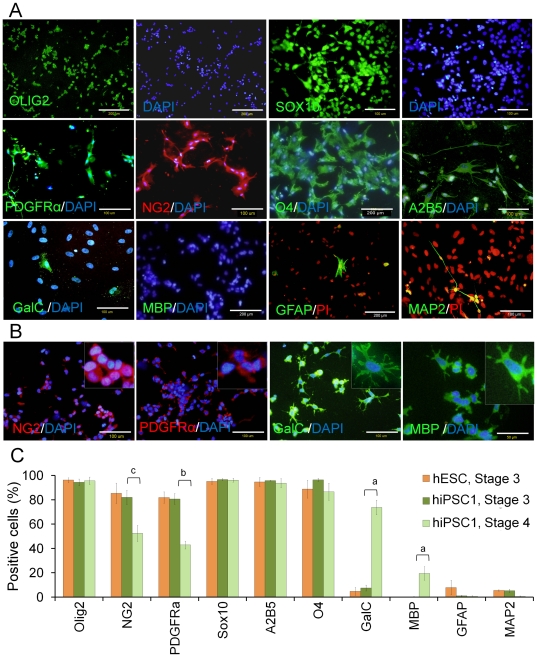
Immunofluorescence staining of differentiated oligodendrocyte lineage cells from hiPSCs and hESCs. (A) The representative pictures of Olig2, Sox10, PDGFRα, NG2, O4, A2B5, GalC, MBP, GFAP, and MAP2 expression in hiPSC1-derived OPs. Oligodendrocyte lineage markers exhibit high levels of expression in the OP stage, while the astrocyte cell protein GFAP and the neuronal protein MAP2 are minimally expressed (A). The removal of EGF from the hiPSC1-derived OPs medium results in down-regulation of NG2 and PDGFRα and up-regulation of GalC and MBP, as shown in (B). The percentage of positive cells at stage 3 and 4 for hiPSC1-derived oligodendrocyte lineage cells and hESC-derived OPs at stage 3 for some markers (C). (c) p<0.05; (b) p<0.01; (a) p<0.001. Error bar: SEM.

We evaluated the OPs (stage 3) and differentiated immature oligodendrocytes (stage 4) at the cellular level by immunofluorescence staining (Figure 3). Random field counting of immunofluorescence images showed that the majority of hiPSC-derived OPs (>90%) expressed related oligodendrocyte lineage markers (Figure 3) including OLIG2 (94.37±2.69%), SOX10 (96.61±0.80%), A2B5 (95.74±0.22%) and O4 (96.46±1.21%). Immunofluorescence in hiPSC-derived OPs was also positive for NG2 (82.04±5.88%) and PDGFRα (80.72±4.43%). However, the number of cells positive for these OP markers significantly decreased to 52.41±6.55% (p<0.05) and 42.87±3.19% (p<0.01) in stage 4, respectively (Figure 3 B, C). The number of counted cells for each antibody has presented in Table S1. PDGFRα, A2B5 and NG2 are membrane epitopes typically expressed in OPs [40], [41]. SOX10 is a member of the high-mobility group domain family of DNA binding proteins expressed within OPs, yet it is not expressed in neurons or astrocytes [42]. The Olig2 transcription factor is critical for oligodendrocyte and motor neuron differentiation [43].

After the final differentiation (stage 4), the expression of oligodendrocyte-specific genes such as MBP and GalC increased (GalC: 7.49±2.37% to 73.75±5.97%, p<0.01; MBP: 0.30±0.15% to 19.51±5.56%, p<0.05). MBP is localized to the major dense lines of myelin and confined to the interior of oligodendrocytes [44], [45]. No significant differences were noted among the expressions of OLIG2, SOX10, O4, GFAP, and MAP2 in hiPSC-derived cells in stages 3 and 4 (Figure 3C). O4 is a surface antigen on immature and mature oligodendrocytes [46]. Importantly, the levels of the astrocyte marker GFAP and the neuronal marker MAP2 were the same before and after differentiation [GFAP (1.07±0.42%) and MAP2 (5.26±1.17%)] (Figure 3C). Therefore, the oligodendrocyte lineage was enriched relative to neurons or astrocytes. Additionally, this *in vitro* protocol for oligodendrocyte differentiation of hESCs resulted in efficient induction of oligodendrocytes and their progenitors from hiPSCs.

Similar results in expression were observed in hESCs (Royan H6 line, Figure 3C) and hiPSC8 in RNA and protein levels (Figures S1 and S2). Therefore, we continued our transplantation experiments with hiPSC1-derived OPs.

### Transplantation into the optic chiasm rat model of demyelination

**Figure 4:**
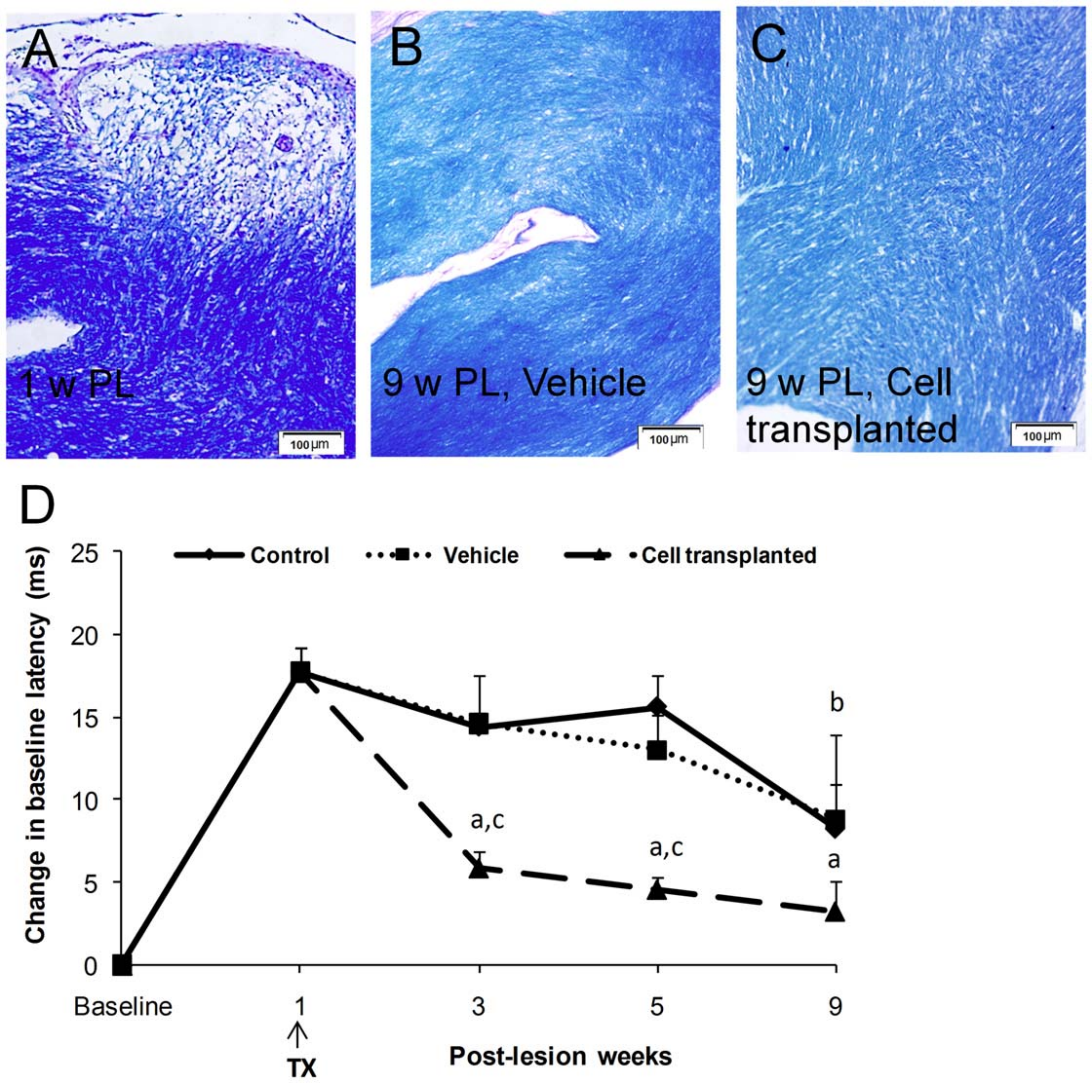
Histological and behavioral assessments following demyelinated chiasm injury and cell transplantation. (A) A longitudinal section of a demyelinated region stained with Luxol fast blue at the first week post-lesion. (B) Representative Luxol fast blue staining of chiasm sections nine weeks' post-lesion in vehicle group. (C) Myelin repair in the optic chiasm using Luxol fast blue in representative sections eight weeks post-transplantation with hiPSC1-derived OPs. (D) Functional evaluation of the optic apparatus using VEP recording in animal models. Changes in the baseline latency of the P1 wave following local injection of hiPSC1-derived OPs in the transplant group are shown along with those of the medium (vehicle) and control groups. The arrow shows the time of cell or medium injection. b: p<0.01 and a: p<0.001 vs. week 1, c: p<0.01 vs. vehicle treated and control groups in the same week. Error bar: SEM. Tx: transplantation. PL: post-lesion.

The amounts of demyelination and remyelination were histologically evaluated using Luxol fast blue myelin-specific staining one, five and nine weeks post-lesion. Extensive demyelination was demonstrated one week post-lesion (Figure 4A). Four and eight weeks after transplantation of hiPSC1-derived OPs an obvious remyelination was seen, while less remyelination was seen in control chiasms (Figure 4B and C).

Improvement in remyelination of the optic chiasm and nerves was functionally evaluated using VEP recording from the skull. Changes in the P1 wave-latency was assessed in the control, vehicle and cell transplanted animals (Figure 4D). While low levels of endogenous recovery of P1-latency were observed in the control and vehicle-treated animals eight weeks post-lesion, cell grafting reduced the elevated latency relative to the control by one week post-transplantation, and this reduction persisted until eight weeks post-transplantation.

**Figure 5:**
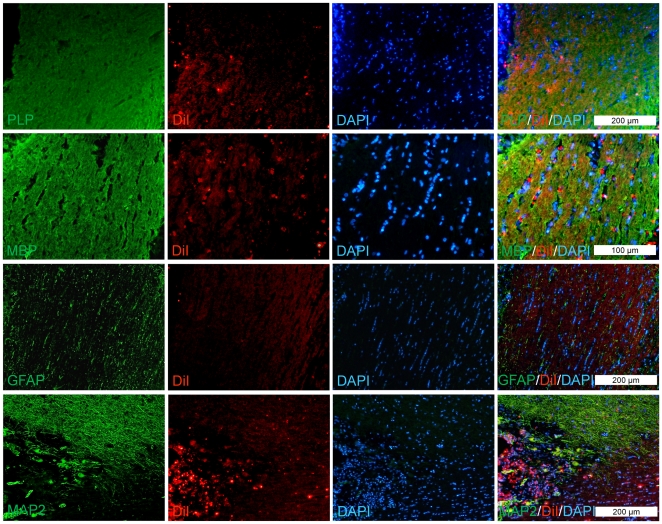
Immunofluorescence staining of transplanted cells. The hiPSC1-derived OPs were labeled with DiI before transplantation into chiasms and immunostained with antibodies against PLP, MBP, GFAP, or MAP2 eight weeks after transplantation. The majority of DiI-labeled cells showed PLP and/or MBP reactivity, and a few of them were GFAP+ and/or MAP2+.

Eight weeks after transplantation, tracing of DiI-labeled cells showed that the transplanted cells survived and integrated within the chiasm (Figure 5). Double labeling for DiI and PLP, MBP, GFAP, or MAP2 showed that the majority of the grafted cells were PLP+ and/or MBP+, which demonstrated differentiation of hiPSC1-derived OPs into mature oligodendrocytes *in vivo* and confirmed remyelination by the transplanted cells (Figure 5). Additionally, a few of the transplanted cells were GFAP+ or MAP2+, which indicated non-astroglial and non-neuronal differentiation of the grafted cells *in vivo*. DiI-unlabeled transplanted cells were used as negative control, eight weeks post-cell transplantation (Figure S3).

Additionally, we did not observe tumor, teratoma or non-neuronal tissue formation in the transplant recipients at four or eight weeks following hiPSC1-derived OPs transplantation.

## Discussion

Several studies have demonstrated generation of oligodendrocyte lineage cells from hESCs with different efficiencies [14]–[23]. Based on these reports, we developed a protocol and applied it to differentiate hiPSC into an oligodendrocyte lineage. Our data showed that our protocol efficiently generated hiPSC-derived OPs *in vitro*. Additionally, the transplantation of these OPs seven days after lysolecithin induced demyelination to the optic chiasm of the rat models resulted in significant functional recovery compared to the control and vehicle groups.

The method used to differentiate hiPSCs toward an oligodendrocytic cell lineage was based on sequential induction at proper time intervals by introducing appropriate induction and growth factors. This method has previously been used to generate OPs from hESC (Hatch et al., 2009).

Our hiPSCs were induced into an oligodendrocyte lineage by EB formation following the plating of yellow spheres that were generated in the presence of RA in GRM. Bipolar and proliferating hiPSC-derived OPs appeared following migration of cells from yellow spheres and became complex ramified oligodendrocytes after growth factors were withdrawn. Thyroid hormone was needed to ensure oligodendrocyte survival and to initiate the differentiation of OPs into mature oligodendrocytes [47].

Moreover, our quantitative real-time PCR and immunostaining data has shown that hiPSCs share similarities in gene expression patterns with hESCs during differentiation to oligodendrocytes. The percentage of antigen-expressing cells measured by immunofluorescence staining exhibited no significant difference between hiPSC-derived OPs and hESC-derived OPs. OP specific markers such as OLIG2, A2B5, SOX10, NG2, PDGFRα, and O4 were expressed in the majority of cells. However, later oligodendrocyte markers, such as GalC and MBP, were expressed in few cells. Contamination with other neural lineage types was low, as few of the cells were positive for GFAP and MAP2 at the OP step. Additionally, the expression of pluripotent genes was strongly down-regulated. We continued to culture adherent hiPSC-derived OPs by trypsinization during several passages (results not shown) using a method similar to one previously described for expandable hESC-derived OPs [48]. These findings demonstrated that a population of pure hiPSC-derived OPs was generated in this study as well as a population of pure hESC-derived OPs.

Furthermore, developmental studies have demonstrated that GalC (70%) and MBP (20%) begin to express in both immature oligodendrocytes and mature oligodendrocytes [49], [50]. These results indicate that hiPSC-derived OPs can differentiate into more mature oligodendrocytes, but our conditions were insufficient to achieve full maturation *in vitro,* which may require other parameters such as cell contact with other types of CNS cells.

This study has assessed the therapeutic effects of transplanted hiPSC-derived OPs in toxically-induced demyelination of the rat chiasm. The production of animal models with CNS white matter locally demyelinated by toxic materials such as lysolecithin provides a good tool for assessing the therapeutic effects of transplanted oligodendrocyte cells. Lysolecithin in applied concentration is toxic and particularly affects myelin-producing cells [51], [52] and can therefore be used to produce local, acute demyelination in the CNS [34]. Cells were transplanted one week after and in the same manner as the lysolecithin injection. Our histology results demonstrated that demyelination occurred in the chiasm one week after the lysolecithin injection. Luxol fast blue staining of chiasm sections four and/or eight weeks after hiPSC-derived OPs transplantation showed increased staining and remyelinating axons. The engrafted OPs survived, migrated and integrated into the host tissue at week's post-cell transplantation. Functional changes to the optic nerve were recorded with VEP measurements. It was also shown that hiPSC-derived OPs promoted recovery in transplanted rats compared to vehicle-injected and control rats. This improvement was observed particularly in the first week after cell transplantation, which indicates the short-term effectiveness of this transplantation. Immunohistofluorescence analysis of the grafts indicated that the majority of the transplanted hiPSC-derived OPs expressed the MBP protein, demonstrating that OPs derived from hiPSCs completed differentiation after transplantation to the demyelinated chiasm. A few of the cells displayed the astrocyte marker, GFAP in the chiasm of the transplanted rats. It has been shown that transplanted hESC-derived OPs improve the recovery of spinal cord injury in demyelination models [14]–[22]. Recently, it was shown that mouse iPSC-OPs could also become functional remyelinating oligodendrocytes, as demonstrated *in vitro* by co-culture of these cells with neurons and *in vivo* after implantation in the demyelinated corpus callosum of cuprizone-treated mice [26]. Additionally, it was reported that axonal loss in eyes that received concurrent inflammatory activated OPs was lower than axonal loss in control eyes in a glaucoma model [53].

On the other hand, in addition to their differentiation into mature oligodendrocytes following transplantation, hiPSC-derived OPs may also have indirect effects on the host tissue through secretion of anti-inflammatory and tropic molecules. hESC-derived OPs have been shown to express several neurotrophic factors that result in neuronal survival and the regeneration of damaged neurons [16]. Also, the trophic effect of transplanted NPs enhanced myelin regeneration of the host OPs in a chronic cuprizone-induced demyelination model in aged mice [54]. Moreover, we could not identify OCT4-positive cells at the OP generation stage. We did not observe teratoma formation after eight weeks, indicating that no undifferentiated iPSCs were present after our oligodendrocyte differentiation procedure.

To our knowledge, this is the first report on the differentiation of hiPSCs into oligodendrocyte-lineage cells and their transplantation in an animal model. These hiPSC-derived OPs improved myelin repair in lysolecithin-induced demyelinated-rat optic chiasm which reveal the possible application of these patient specific cells in future regenerative biomedicine. However, several issues remain to be solved before hiPSC-derived cells can be safely applied in clinical settings (for reviews, see [3], [4]). For example, recently it has reported that transplantation of undifferentiated iPSCs which derived by either retroviral approach or a episomal approach showed T-cell-dependent immune response in recipient syngeneic mice due to the abnormal expression of antigens following genetic manipulation [55], [56]. Improvements in the production of safe and non-immunogenic hiPSCs will increase the number of applications of iPSC derivatives.

## Materials and Methods

### Pluripotent stem cell culture and differentiation into oligodendrocyte lineage cells

The hiPSC line Royan hiPSC1 and hiPSC8 [28] at passages 29–47 was used in these experiments. Cells were expanded and passaged under feeder-free culture conditions in a hESC medium that contained 100 ng/ml basic fibroblast growth factor (bFGF, Royan Institute) using a previously described technique [29]. The medium was changed every other day until day 7. The differentiation procedure (outlined in Figure 1) was divided into four stages, as described in (Hatch et al., 2009). The hiPSC colonies were exposed to an enzymatic mixture that contained 1 mg/ml collagenase IV (Invitrogen, 17104-019) and 2 mg/ml dispase (Invitrogen, 17105-041) for 7 min at 37°C. The enzymes were then removed, and the colonies were rinsed with Dulbecco's phosphate-buffered saline (D-PBS, Invitrogen, 14190-144). Cell clusters were scraped from the dish after the addition of hESC medium/glial restriction medium (GRM) at a 1∶1 ratio and collected in low-attachment bacteriological dishes (Falcon, 351029). A total of 4 ng/ml bFGF was added to the culture media, and cells were then transferred to an incubator. The GRM consisted of DMEM/F12 (Invitrogen, 21331-020), 2% B27 supplement (Invitrogen, 17504-044), 2 mM L-glutamine (Invitrogen, 25030-024), 10 µg/ml insulin (Sigma-Aldrich, I1882), 10 µg/ml putrescine (Sigma-Aldrich, P6024), 63 ng/ml progesterone (Sigma-Aldrich, P6149), 50 ng/ml sodium selenite (Sigma-Aldrich, S9133), 50 µg/ml holo-transferrin (Sigma-Aldrich, T1408), and 40 ng/ml triiodothyronine (T3; Sigma-Aldrich, T6397). The following day, cell clusters were collected and transferred to hESC medium/GRM containing 4 ng/ml bFGF, 20 ng/ml human epidermal growth factor (EGF; Sigma-Aldrich, E9644) and 10 µM all-trans-retinoic acid (RA; Sigma-Aldrich, R2625). Over the next eight days, the medium was exchanged daily with GRM containing 20 ng/ml EGF and 10 µM RA. After eight days, RA was omitted from the media, and floating clusters were cultured for 18 days during which the medium was changed every other day. At day 28 of the differentiation protocol, yellow clusters were cultured on matrigel- (Sigma-Aldrich, E1270) coated 6 cm tissue culture dishes in the same medium. Clusters adhered to the dishes, and cells were dislodged one week later using 0.05% trypsin/EDTA (Invitrogen, 25300) for 3 min at 37°C. Trypsin was neutralized with DMEM/F12 containing 10% fetal bovine serum (FBS; Hyclone, SH30070) and centrifuged, and the cell pellet was resuspended in GRM plus 20 ng/ml EGF and reseeded on matrigel-coated dishes for seven days. Cells underwent weekly passaging.

OPs were more differentiated on poly-L-Lysine (Sigma-Aldrich, P4707)/laminin-coated plates (Sigma-Aldrich, L2020) (PLL/lam) in the absence of EGF over a three-week period. In some of the experiments, the Royan H6 hESC line [30] at passages 102–127 was used as a control group.

### Real-time reverse transcription PCR

At each stage, cells were collected, and the total RNA was extracted using TRIzol reagent (Invitrogen, 15596-018). DNA was degraded with the use of the DNase I, RNase-free kit (Fermentas, EN0521), whereas RNA was protected using RiboLockTM RNase Inhibitor (Fermentas, E00381). cDNA was synthesized using a RevertAidTM H Minus First Strand cDNA Synthesis kit (Fermentas, K1632). Relative gene expression analysis was performed with real-time PCR. The expression of several genes, including POU5F1 (Oct4), Sox2, Pax-6, Tuj1, Olig2 PDGFRα and a housekeeping gene, β-actin, were analyzed at four stages of the oligodendrocyte differentiation procedure. Real-time PCR used specific primers (Table S2), a 96-well optical reaction plate and the 7500 Real Time PCR system (Applied Biosystems). In each PCR reaction, 1X Power SYBR® Green PCR Master Mix (ABI PRISM, 4368702) was mixed with 12 ng cDNA and specific primers in a total volume of 20 µl. Thermal conditions were the same for all genes, and the annealing temperature was 60°C.

The comparative Ct method, 2^−ΔΔCt^, was used for relative gene expression analysis [31]. All Ct values calculated from the target genes were normalized to β-actin and calibrated using calculations from the undifferentiated pluripotent cells from stage 1. In each experiment, there were at least three independent replicates of each stage, and each replicate included two identical samples.

### Immunofluorescence staining

For immunofluorescence staining, cultured cells were washed with washing buffer (phosphate-buffered saline (PBS) with 0.05% tween-20 (Sigma-Aldrich, P7949), pH 7.4) and fixed with 4% paraformaldehyde (Sigma-Aldrich, P6148) in PBS for 10 min. If necessary, cells were then permeabilized with 0.5% Triton X-100 (Sigma-Aldrich, T8532) for 15 min. Cells were twice washed and blocked in 10% secondary antibody host serum in washing buffer for 30 min at 37°C. Cells were incubated overnight at 4°C with primary antibodies (Table S3) diluted in washing buffer. Cells were washed three times (5 min each) and then incubated with fluorescence-labeled secondary antibodies in washing buffer at room temperature and rinsed three times (5 min each). For negative controls, only the secondary antibody was used (results not shown). Either the fluorescent dye propidium iodide (PI; Fluka, 81845) or 4′,6-diamidino-2-phenylindole (DAPI; Sigma-Aldrich, D-8417) was used for staining nuclei.

Images were obtained on an Olympus IX71 fluorescence microscope with a DP72 digital camera and captured by analySIS LS Starter version 3.2. For cell counting, 10 random images were prepared from different sites on each plate, and fluorescent-stained cells of each marker were counted in each image. The experiment was performed on at least three independent cell cultures for each antibody. The percentage of positive cells was determined as the ratio of positive cells to the total number of counted cells (stained with PI or DAPI) in each experiment.

### Implantation of hiPSC-derived OPs in lysolecithin-induced demyelinated rat chiasm

To evaluate the capacity of hiPSC-derived OPs to restore myelination *in vivo*, cells were implanted in the demyelinated optic chiasm of lysolecithin-injected rat models.

#### Ethics statement

All animal use procedures were in strict accordance with the approval of the Royan Institutional Review Board and Institutional Ethical Committee under the approval ID of J/90/1397.

#### Animals

Adult male Sprague Dawley rats (240–280 g) were obtained from Razi Institute (Karaj, Iran). Rats were maintained in temperature-controlled rooms with a 12/12 h light/dark cycle and 50–55% humidity and had free access to food and water.

#### Preparation of cells for transplantation

For identification after transplantation, the cells were prelabeled with the plasma membrane marker, red fluorescence-CellTracker CM-DiI (chloromethyl benzyl-amide derivatives of 1,1-dioctadecyl-3,3,3′,3′ tetramethylindocarbocyanine perchlorate; Invitrogen, C7001), which allowed *in vivo* identification of grafted cells. Cells were incubated in 5 µg/ml CM-DiI in GRM medium for 20 min in the incubator. Cells were then washed three times (3×5 min) in D-PBS (Invitrogen, 14190-144). The viability of cells before transplantation was determined by Trypan blue exclusion dye assay to be greater than 95%.

#### Surgical procedure and cell transplantation

The animals were anesthetized with intraperitoneal (i.p.) injections of 50 mg/kg ketamine and 5 mg/kg xylazine and placed in a stereotaxic instrument. A 30-gauge stainless steel needle was used to inject lysolecithin into the chiasm (anterior-posterior: 0.1 mm from the bregma, lateral: 0 mm and vertical: 4.5 mm from the dura) [32]. All animals were administered 2 µl of 1% lysolecithin (Sigma-Aldrich, L1381) at day zero.

This treatment leads to demyelination of axons after one week. However, spontaneous remyelination will occur over longer durations due to endogenous OPs recruited from the adjacent tissues [33]–[35]. Therefore, one week after lysolecithin administration, rats were divided randomly into three groups: the cell-transplanted group (n = 8) received 200,000 hiPSC-derived OPs in 2 µl medium; the vehicle-transplanted group (n = 8) received 2 µl medium; and a control group (n = 8) received no cells and no vehicle. Cells were transplanted into the chiasm using a 25 µl Hamilton syringe connected to a 30 gauge needle. A microinjection system (Stoelting, Illinois, USA) infused the suspension at a rate of 0.5 µl/minute. After grafting, the needle remained in place for 1 minute to minimize cell reflux into the needle track. The animals were then housed in standard rat cages at a temperature of 27°C. The rats were immune-suppressed by addition of 210 mg/l, cyclosporine-A (Sandimmune, Novartis Pharmaceuticals, East Hanover, NJ, USA) to the drinking water two days before cell transplantation [36], [37]. The animals then remained on the same immunosuppressant throughout the experiment. Serum levels of cyclosporine were measured in a random sample of animals at termination and the average concentration was 1750±36 ng/ml (n = 2, mean±SEM).

#### Visual evoked potential (VEP) recording

Functional changes in the optic nerve were electrophysiologically studied using VEP recording before lysolecithin injection and at 1st, 3nd, 5th and 9th weeks post-lesion in all groups. VEP can provide important diagnostic information regarding the functional integrity of the visual system [38]. VEP recording was performed using a previously described technique [35]. Briefly, two stainless steel screws were placed into the skull to serve as electrodes. The dura was kept intact to minimize cortical damage. The “reference” electrode was placed 5 mm anterior to the bregma and 1.5 mm right of midline, and the “record” electrode was placed 7 mm caudal to the bregma and 3 mm right of midline. Light stimulation was delivered by a general evoked response stimulator (WSI, Iran) 300 times at a frequency of 0.5 Hz. Responses were amplified using an amplifier (Iso-80, UK). The amplified waveforms were averaged. In each VEP recording, the latencies between the light flash and the first positive peak were studied.

#### Histochemical and immunohistofluorescence analyses

To study the extent of demyelination, the animals were sacrificed using a lethal dose of anesthesia one week after lysolecithin injection and five and nine weeks post-lesion in cell transplanted and vehicle treated groups (n = 3 per group). The chiasms were immediately stored and post-fixed in 4% paraformaldehyde for 24 h. Some chiasms were processed and embedded in paraffin. Slices were stained with Luxol fast blue to stain myelin and nuclei were counterstained with cresyl violet. Tumor formation in the transplanted animals was assessed by H&E staining.

To follow-up the fate of DiI-labeled transplanted cells, after fixation in cryoprotected 30% sucrose in PBS at 4°C for 24 h in a cryostat, some chiasms (n = 3 per group) were processed for immunohistofluorescence analysis of the cell implants. Tissue was frozen in Jung tissue freezing medium (Leica Microsystems) at −30°C and sectioned with a cryotome into pieces 8 µm thick (Leica Microsystems, CM 1850). The sections were permeabilized with 0.04% Triton X-100 and blocked with 10% normal goat serum and 0.5% BSA (Sigma-Aldrich, A3311) in PBS for 1 h. Primary antibodies against PLP, MBP, GFAP, MAP2 (Table S3) were applied overnight at room temperature. Sections were washed, and the appropriate secondary antibodies, which were conjugated with fluorescent dye (Table S3), were applied for 1 h at 37°C. Sections were washed and counterstained with the nuclear dye DAPI. Each protein was evaluated in three chiasms. Images were obtained on an Olympus BX51 fluorescence microscope with a DP72 digital camera and captured by analySIS LS Starter version 3.2.

### Statistical analysis

The data are expressed as mean ± SEM. A difference between groups was considered statistically significant if p<0.05. For immunofluorescence staining, an unpaired, two-tailed t-test was used to assess statistically significant differences. For qRT-PCR, randomization tests using REST 2009 V2.0.13 software were applied to indicate statistically significant differences between stage 1 and the remaining stages [39]. All quantitative data and any significant differences in the latency of the VEPs of each group were tested using a mixed-model ANOVA with a repeated factor (time) and a non-repeated factor (group). One-way ANOVA followed by post-test LSD was performed for each group.

## Supporting Information

Figure S1 **Relative gene expression using real-time PCR for hiPSC8 oligodendroglial-lineage cell differentiation.** Log_2_ changes in expression are reported. In real-time PCR, sampling was done in four different stages during the differentiation protocol. The primers of *OCT4* and *SOX2* were used for pluripotency, *TUJ1* for neural lineage, and *PDGFRα* for oligodendrocyte lineage differentiation. Obviously, pluripotent specific genes decreased during the differentiation of hiPSC8 to oligodendrocyte when compared to stage 1. The expression levels of PDGFRα were increased; however its expression deceased in stage 4. b: p<0.01, a: p<0.001; Error bar: SEM.
(TIF)

Figure S2 **Immunofluorescence staining of differentiated oligodendrocyte lineage cells from hiPSC8.** (A) The representative pictures of SOX10, PDGFRα, NG2, O4, A2B5, GalC, GFAP, and MAP2 expression in hiPSC8-OPs. Oligodendrocyte lineage markers showed high levels of expression in the OP stage whereas, the astrocyte cell protein, GFAP and neuronal protein MAP2 were minimally expressed (A). The down-regulation of NG2 (p<0.05) and PDGFRα (p<0.05) and up-regulation of GalC (p<0.05) as shown in (B) were observed following removal of EGF. The percentage of positive cells at stage 3 for some markers compared with expression in stage 4 (C). (c) p<0.05. Error bar: SEM. Scale bars in part (A) are 100 µm.
(TIF)

Figure S3 **Negative control for DiI labeling.** DiI unlabeled transplanted cells were used as negative control, 9 weeks post lesioning.
(TIF)

Table S1 **The number of counted cells counterstained with DAPI or PI in different groups.**
(DOC)

Table S2 **List of primers used for Real-Time PCR analysis.**
(DOC)

Table S3 **List of antibodies used in this study.**
(DOC)

## References

[1] Martino G, Franklin RJ, Van Evercooren AB, Kerr DA (2010). Stem cell transplantation in multiple sclerosis: current status and future prospects.. Nat Rev Neurol.

[2] Takahashi K, Yamanaka S (2006). Induction of pluripotent stem cells from mouse embryonic and adult fibroblast cultures by defined factors.. Cell.

[3] Stadtfeld M, Hochedlinger K (2010). Induced pluripotency: history, mechanisms, and applications.. Genes Dev.

[4] Gonzalez F, Boue S, Belmonte JC (2011). Methods for making induced pluripotent stem cells: reprogramming a la carte.. Nat Rev Genet.

[5] Plath K, Lowry WE (2011). Progress in understanding reprogramming to the induced pluripotent state.. Nat Rev Genet.

[6] Lister R, Pelizzola M, Kida YS, Hawkins RD, Nery JR (2011). Hotspots of aberrant epigenomic reprogramming in human induced pluripotent stem cells.. Nature.

[7] Zhu H, Lensch MW, Cahan P, Daley GQ (2011). Investigating monogenic and complex diseases with pluripotent stem cells.. Nat Rev Genet.

[8] Chambers SM, Fasano CA, Papapetrou EP, Tomishima M, Sadelain M (2009). Highly efficient neural conversion of human ES and iPS cells by dual inhibition of SMAD signaling.. Nat Biotechnol.

[9] Nemati S, Hatami M, Kiani S, Hemmesi K, Gourabi H (2010). Long-term Self-Renewable Feeder-Free Human Induced Pluripotent Stem Cell-derived Neural Progenitors.. Stem Cells Dev: [Epub ahead of print].

[10] Onorati M, Camnasio S, Binetti M, Jung CB, Moretti A (2010). Neuropotent self-renewing neural stem (NS) cells derived from mouse induced pluripotent stem (iPS) cells.. Mol Cell Neurosci.

[11] Tsuji O, Miura K, Okada Y, Fujiyoshi K, Mukaino M (2010). Therapeutic potential of appropriately evaluated safe-induced pluripotent stem cells for spinal cord injury.. Proc Natl Acad Sci U S A.

[12] Crocker SJ, Bajpai R, Moore CS, Frausto RF, Brown GD (2011). Intravenous Administration of Human ES-derived Neural Precursor Cells Attenuates Cuprizone-induced CNS Demyelination.. Neuropathol Appl Neurobiol.

[13] Erceg S, Ronaghi M, Oria M, Rosello MG, Arago MA (2010). Transplanted oligodendrocytes and motoneuron progenitors generated from human embryonic stem cells promote locomotor recovery after spinal cord transection.. Stem Cells.

[14] Cloutier F, Siegenthaler MM, Nistor G, Keirstead HS (2006). Transplantation of human embryonic stem cell-derived oligodendrocyte progenitors into rat spinal cord injuries does not cause harm.. Regen Med.

[15] Faulkner J, Keirstead HS (2005). Human embryonic stem cell-derived oligodendrocyte progenitors for the treatment of spinal cord injury.. Transpl Immunol.

[16] Zhang YW, Denham J, Thies RS (2006). Oligodendrocyte progenitor cells derived from human embryonic stem cells express neurotrophic factors.. Stem Cells Dev.

[17] Hu BY, Zhang SC (2009). Differentiation of spinal motor neurons from pluripotent human stem cells.. Nat Protoc.

[18] Hu BY, Du ZW, Li XJ, Ayala M, Zhang SC (2009). Human oligodendrocytes from embryonic stem cells: conserved SHH signaling networks and divergent FGF effects.. Development.

[19] Hu Z, Li T, Zhang X, Chen Y (2009). Hepatocyte growth factor enhances the generation of high-purity oligodendrocytes from human embryonic stem cells.. Differentiation.

[20] Keirstead HS, Nistor G, Bernal G, Totoiu M, Cloutier F (2005). Human embryonic stem cell-derived oligodendrocyte progenitor cell transplants remyelinate and restore locomotion after spinal cord injury.. J Neurosci.

[21] Nistor GI, Totoiu MO, Haque N, Carpenter MK, Keirstead HS (2005). Human embryonic stem cells differentiate into oligodendrocytes in high purity and myelinate after spinal cord transplantation.. Glia.

[22] Sharp J, Frame J, Siegenthaler M, Nistor G, Keirstead HS (2010). Human embryonic stem cell-derived oligodendrocyte progenitor cell transplants improve recovery after cervical spinal cord injury.. Stem Cells.

[23] Kerr CL, Letzen BS, Hill CM, Agrawal G, Thakor NV (2010). Efficient differentiation of human embryonic stem cells into oligodendrocyte progenitors for application in a rat contusion model of spinal cord injury.. Int J Neurosci.

[24] Hu BY, Du ZW, Zhang SC (2009). Differentiation of human oligodendrocytes from pluripotent stem cells.. Nat Protoc.

[25] Tokumoto Y, Ogawa S, Nagamune T, Miyake J (2010). Comparison of efficiency of terminal differentiation of oligodendrocytes from induced pluripotent stem cells versus embryonic stem cells in vitro.. J Biosci Bioeng.

[26] Czepiel M, Balasubramaniyan V, Schaafsma W, Stancic M, Mikkers H (2011). Differentiation of induced pluripotent stem cells into functional oligodendrocytes.. Glia.

[27] Hatch MN, Nistor G, Keirstead HS (2009). Derivation of high-purity oligodendroglial progenitors.. Methods Mol Biol.

[28] Totonchi M, Taei A, Seifinejad A, Tabebordbar M, Rassouli H (2010). Feeder- and serum-free establishment and expansion of human induced pluripotent stem cells.. Int J Dev Biol.

[29] Pakzad M, Totonchi M, Taei A, Seifinejad A, Hassani SN (2010). Presence of a ROCK inhibitor in extracellular matrix supports more undifferentiated growth of feeder-free human embryonic and induced pluripotent stem cells upon passaging.. Stem Cell Rev.

[30] Baharvand H, Ashtiani SK, Taee A, Massumi M, Valojerdi MR (2006). Generation of new human embryonic stem cell lines with diploid and triploid karyotypes.. Dev Growth Differ.

[31] Livak KJ, Schmittgen TD (2001). Analysis of relative gene expression data using real-time quantitative PCR and the 2(-Delta Delta C(T)) Method.. Methods.

[32] Paxinos G, Watson C (2007). The rat brain in stereotaxic coordinates.. Academic Press/Elsevier 6th ed.

[33] Hall SM (1972). The effect of injections of lysophosphatidyl choline into white matter of the adult mouse spinal cord.. J Cell Sci.

[34] Woodruff RH, Franklin RJ (1999). The expression of myelin protein mRNAs during remyelination of lysolecithin-induced demyelination.. Neuropathol Appl Neurobiol.

[35] Mozafari S, Sherafat MA, Javan M, Mirnajafi-Zadeh J, Tiraihi T (2010). Visual evoked potentials and MBP gene expression imply endogenous myelin repair in adult rat optic nerve and chiasm following local lysolecithin induced demyelination.. Brain Res.

[36] Idelson M, Alper R, Obolensky A, Ben-Shushan E, Hemo I (2009). Directed differentiation of human embryonic stem cells into functional retinal pigment epithelium cells.. Cell Stem Cell.

[37] Vugler A, Carr AJ, Lawrence J, Chen LL, Burrell K (2008). Elucidating the phenomenon of HESC-derived RPE: anatomy of cell genesis, expansion and retinal transplantation.. Exp Neurol.

[38] Hudnell HK, Boyes WK, Otto DA (1990). Rat and human visual-evoked potentials recorded under comparable conditions: a preliminary analysis to address the issue of predicting human neurotoxic effects from rat data.. Neurotoxicol Teratol.

[39] Pfaffl MW, Horgan GW, Dempfle L (2002). Relative expression software tool (REST) for group-wise comparison and statistical analysis of relative expression results in real-time PCR.. Nucleic Acids Res.

[40] Zhou Q, Wang S, Anderson DJ (2000). Identification of a novel family of oligodendrocyte lineage-specific basic helix-loop-helix transcription factors.. Neuron.

[41] Reubinoff BE, Itsykson P, Turetsky T, Pera MF, Reinhartz E (2001). Neural progenitors from human embryonic stem cells.. Nat Biotechnol.

[42] Kuhlbrodt K, Herbarth B, Sock E, Hermans-Borgmeyer I, Wegner M (1998). Sox10, a novel transcriptional modulator in glial cells.. J Neurosci.

[43] Takebayashi H, Yoshida S, Sugimori M, Kosako H, Kominami R (2000). Dynamic expression of basic helix-loop-helix Olig family members: implication of Olig2 in neuron and oligodendrocyte differentiation and identification of a new member, Olig3.. Mech Dev.

[44] Zhang SC (2001). Defining glial cells during CNS development.. Nat Rev Neurosci.

[45] Zhang SC, Wernig M, Duncan ID, Brustle O, Thomson JA (2001). In vitro differentiation of transplantable neural precursors from human embryonic stem cells.. Nat Biotechnol.

[46] Cai Z, Pang Y, Xiao F, Rhodes PG (2001). Chronic ischemia preferentially causes white matter injury in the neonatal rat brain.. Brain Res.

[47] Durand B, Raff M (2000). A cell-intrinsic timer that operates during oligodendrocyte development.. Bioessays.

[48] Brolen G, Sivertsson L, Bjorquist P, Eriksson G, Ek M (2010). Hepatocyte-like cells derived from human embryonic stem cells specifically via definitive endoderm and a progenitor stage.. J Biotechnol.

[49] Espinosa-Jeffrey A, Wakeman DR, Kim SU, Snyder EY, de Vellis J (2009). Culture system for rodent and human oligodendrocyte specification, lineage progression, and maturation.. Curr Protoc Stem Cell Biol Chapter 2: Unit 2D.

[50] Buchet D, Baron-Van Evercooren A (2009). In search of human oligodendroglia for myelin repair.. Neurosci Lett.

[51] Allt G, Ghabriel MN, Sikri K (1988). Lysophosphatidyl choline-induced demyelination. A freeze-fracture study.. Acta Neuropathol.

[52] Dousset V, Brochet B, Vital A, Gross C, Benazzouz A (1995). Lysolecithin-induced demyelination in primates: preliminary in vivo study with MR and magnetization transfer.. AJNR Am J Neuroradiol.

[53] Bull ND, Irvine KA, Franklin RJ, Martin KR (2009). Transplanted oligodendrocyte precursor cells reduce neurodegeneration in a model of glaucoma.. Invest Ophthalmol Vis Sci.

[54] Einstein O, Friedman-Levi Y, Grigoriadis N, Ben-Hur T (2009). Transplanted neural precursors enhance host brain-derived myelin regeneration.. J Neurosci.

[55] Gore A, Li Z, Fung HL, Young JE, Agarwal S (2011). Somatic coding mutations in human induced pluripotent stem cells.. Nature.

[56] Zhao T, Zhang ZN, Rong Z, Xu Y (2011). Immunogenicity of induced pluripotent stem cells.. Nature.

