# Supplementary figures and images for: Human Induced Pluripotent Stem Cells Differentiation into Oligodendrocyte Progenitors and Transplantation in a Rat Model of Optic Chiasm Demyelination
Source: PLoS One. 2011 Nov 18;6(11):e27925. doi: 10.1371/journal.pone.0027925 (PMC3220701; doi:10.1371/journal.pone.0027925)

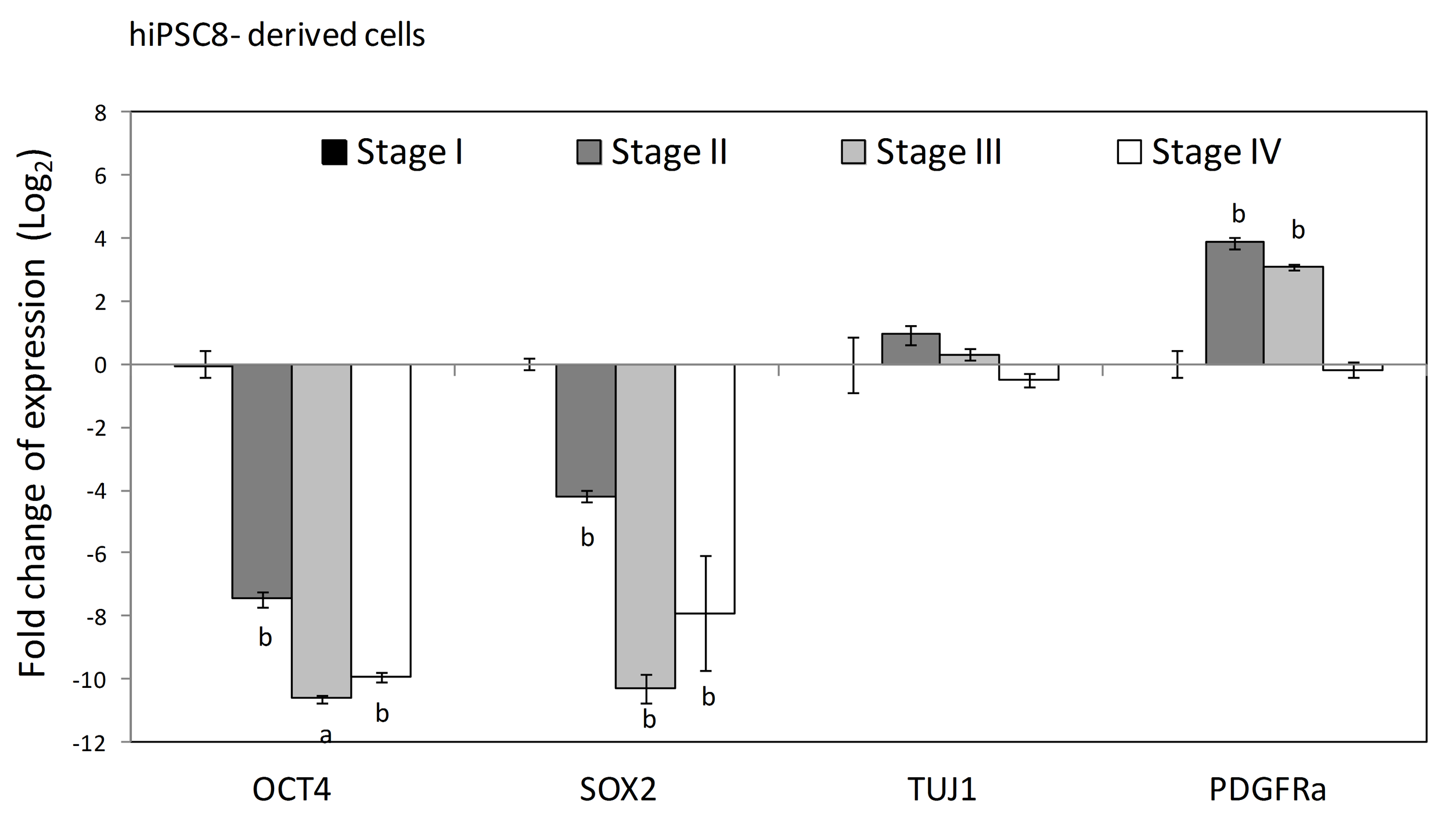

Supplement: Figure S1 — Relative gene expression using real-time PCR for hiPSC8 oligodendroglial-lineage cell differentiation. Log2 changes in expression are reported. In real-time PCR, sampling was done in four different stages during the differentiation protocol. The primers of OCT4 and SOX2 were used for pluripotency, TUJ1 for neural lineage, and PDGFRα for oligodendrocyte lineage differentiation. Obviously, pluripotent specific genes decreased during the differentiation of hiPSC8 to oligodendrocyte when compared to stage 1. The expression levels of PDGFRα were increased; however its expression deceased in stage 4. b: p<0.01, a: p<0.001; Error bar: SEM. (TIF) [file pone.0027925.s001.tif]

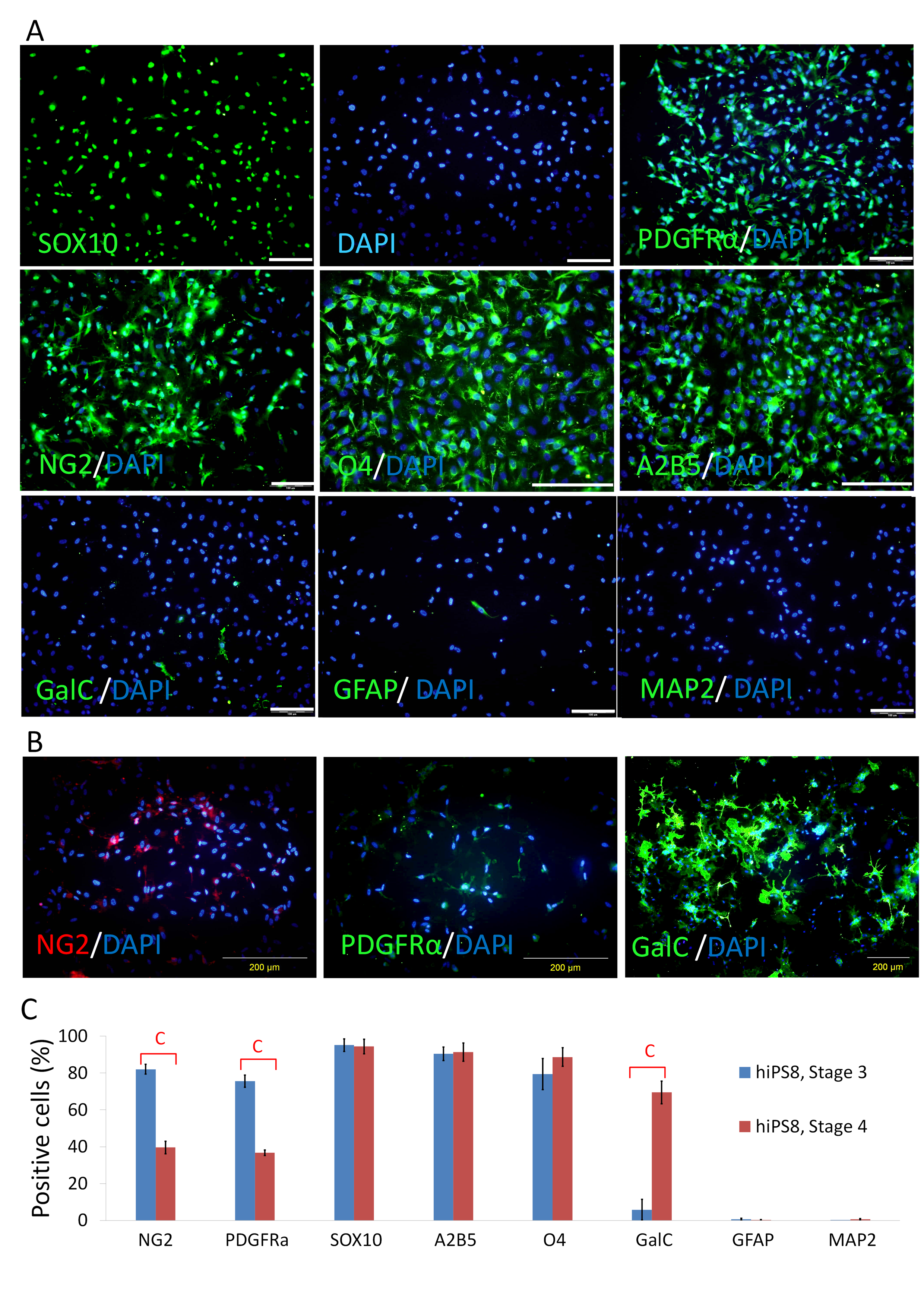

Supplement: Figure S2 — Immunofluorescence staining of differentiated oligodendrocyte lineage cells from hiPSC8. (A) The representative pictures of SOX10, PDGFRα, NG2, O4, A2B5, GalC, GFAP, and MAP2 expression in hiPSC8-OPs. Oligodendrocyte lineage markers showed high levels of expression in the OP stage whereas, the astrocyte cell protein, GFAP and neuronal protein MAP2 were minimally expressed (A). The down-regulation of NG2 (p<0.05) and PDGFRα (p<0.05) and up-regulation of GalC (p<0.05) as shown in (B) were observed following removal of EGF. The percentage of positive cells at stage 3 for some markers compared with expression in stage 4 (C). (c) p<0.05. Error bar: SEM. Scale bars in part (A) are 100 µm. (TIF) [file pone.0027925.s002.tif]

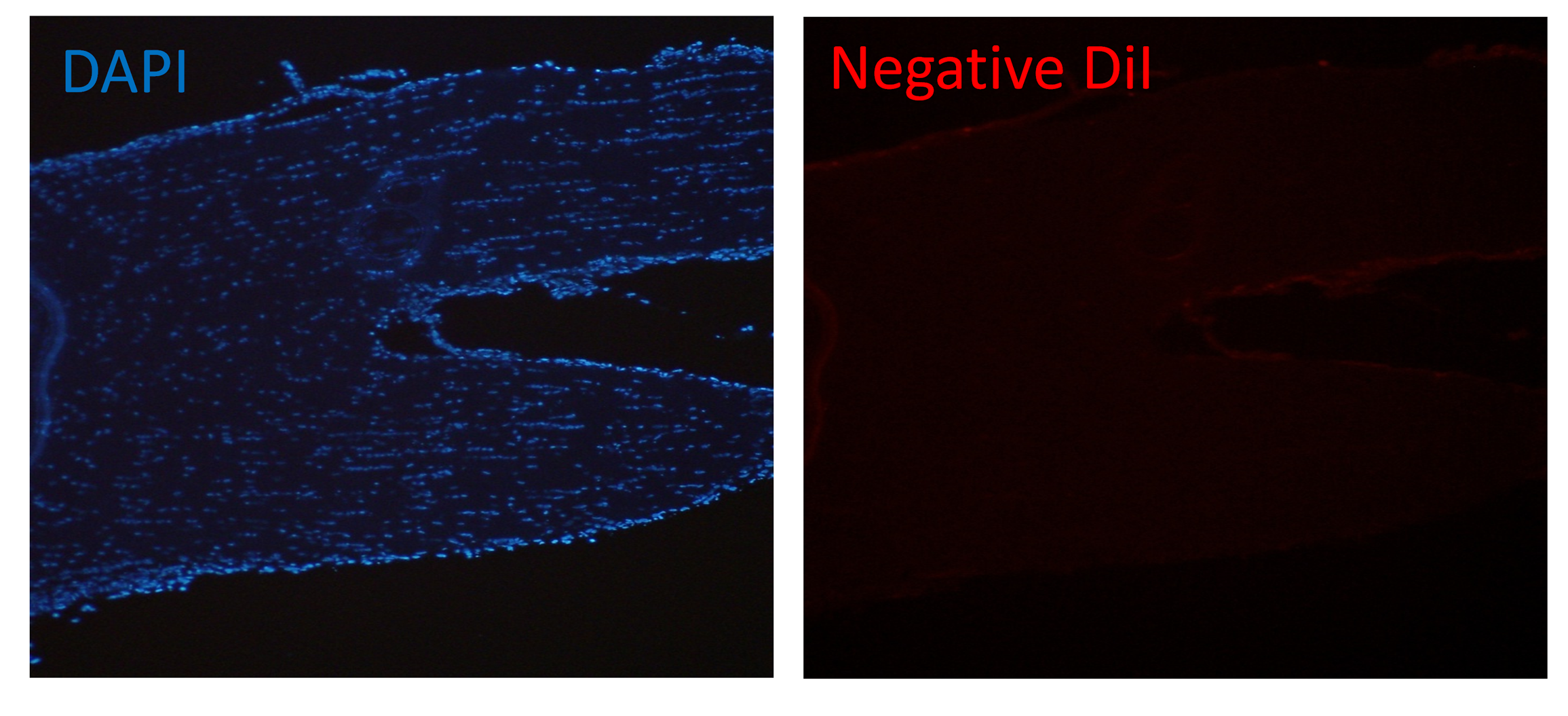

Supplement: Figure S3 — Negative control for DiI labeling. DiI unlabeled transplanted cells were used as negative control, 9 weeks post lesioning. (TIF) [file pone.0027925.s003.tif]
