# Supplementary material for: Human Induced Pluripotent Stem Cells Differentiation into Oligodendrocyte Progenitors and Transplantation in a Rat Model of Optic Chiasm Demyelination
Source: PLoS One. 2011 Nov 18;6(11):e27925. doi: 10.1371/journal.pone.0027925 (PMC3220701; doi:10.1371/journal.pone.0027925)
Supplement: Table S1 — The number of counted cells counterstained with DAPI or PI in different groups. (DOC) [file pone.0027925.s004.doc]

**Table S1.** The number of counted cells counterstained with DAPI or PI in different groups.

| **Counted cells in** | **A2B5** | **O4** | **MBP** | **Olig2** | **NG2** | **PDGFRα** | **Sox10** | **GalC** | **GFAP** | **MAP2** |
| --- | --- | --- | --- | --- | --- | --- | --- | --- | --- | --- |
| **hESC (Royan H6)-derived OPs** | 2128 | 1341 | 1355 | 1221 | 1260 | 1328 | 1740 | 1195 | 1841 | 1582 |
| **hiPSC1-derived OPs** | 1758 | 980 | 1476 | 2041 | 3819 | 2675 | 4203 | 3917 | 2082 | 2655 |
| **hiPSC1- differentiated immature oligodendrocytes** | 1426 | 2448 | 1641 | 2034 | 1850 | 1238 | 2190 | 1747 | 1105 | 1173 |
| **hiPSC8-derived OPs** | 3490 | 1805 | ND | ND | 1782 | 3378 | 1735 | 2086 | 2011 | 1903 |
| **hiPSC8- differentiated immature oligodendrocytes** | 1936 | 1893 | ND | ND | 1932 | 1666 | 1876 | 3892 | 1535 | 1404 |

ND: not determined.
