# Supplementary material for: Human Induced Pluripotent Stem Cells Differentiation into Oligodendrocyte Progenitors and Transplantation in a Rat Model of Optic Chiasm Demyelination
Source: PLoS One. 2011 Nov 18;6(11):e27925. doi: 10.1371/journal.pone.0027925 (PMC3220701; doi:10.1371/journal.pone.0027925)
Supplement: Table S2 — List of primers used for Real-Time PCR analysis. (DOC) [file pone.0027925.s005.doc]

**Table S2.** List of primers used for Real-Time PCR analysis.

| **Gene** | **Primer Sequence** | **Accession No.** |
| --- | --- | --- |
| ß-ACTIN | F: 5'- ttg ccg aca gga tgc aga agg a -3'  R: 5'- agg tgg aca gcg agg cca gga t -3' | NM_001101.2 |
| POU5F1 (OCT4) | F: 5'- gac agg ggg agg gga gga gct agg -3'  R: 5'- ctt ccc tcc aac cag ttg ccc caa ac -3' | NM_002701.4 |
| SOX2 | F: 5'- ggg aaa tgg gag ggg tgc aaa aga gg -3'  R: 5'- ttg cgt gag tgt gga tgg gat tgg tg -3' | NM_003106 |
| TUJ1 | F: 5'- gta tcc cga ccg cat cat -3'  R: 5'- tct cat ccg tgt tct cca -3' | NM_006086 |
| PAX6 | F: 5'- cgg ttt cct cct tca cat -3'  R: 5'- atc ata act ccg ccc at -3' | NM_000280.3 |
| PDGFRα | F: 5'- tac act tgc tat tac aac cac a -3'  R: 5'- atc ctc cac gat gac taa at -3' | NM_006206.3 |
| OLIG2 | F: 5'- cga ctc atc ttt cct tct cta a -3'  R: 5'- cgc act tac ctc atc att g -3' | NM_005806.2 |
