# Supplementary material for: Human Induced Pluripotent Stem Cells Differentiation into Oligodendrocyte Progenitors and Transplantation in a Rat Model of Optic Chiasm Demyelination
Source: PLoS One. 2011 Nov 18;6(11):e27925. doi: 10.1371/journal.pone.0027925 (PMC3220701; doi:10.1371/journal.pone.0027925)
Supplement: Table S3 — List of antibodies used in this study. (DOC) [file pone.0027925.s006.doc]

**Table S3.** List of antibodies used in this study.

| **Antibody** | Isotype | **Dilution** | **Company** | **Cat.NO** |
| --- | --- | --- | --- | --- |
| OLIG2 | Goat IgG | 1 : 100 | Santa Cruz | sc-19967 |
| NG2 | Rabbit poly | 1 : 200 | Millipore | AB5320 |
| PDGFRα | Mouse IgG | 1 : 50 | Santa Cruz | sc-21789 |
| A2B5 | Mouse IgM | 1 : 500 | Sigma-Aldrich | A-8229 |
| O4 | Mouse IgM | 1 : 500 | Sigma-Aldrich | O-7139 |
| SOX10 | Mouse IgG | 1 : 200 | R&D Systems | MAB2864 |
| GalC | Mouse IgG | 1 : 200 | Chemicon | MAB342 |
| MBP | Rat IgG | 1 : 100 | abcam | ab7349 |
| PLP | Rabbit polyclonal | 1 : 100 | abcam | ab28486 |
| GFAP | Mouse IgG | 1 : 200 | Sigma-Aldrich | G-3893 |
| MAP-2 | Mouse IgG | 1 : 200 | Sigma-Aldrich | M-1406 |
| Goat anti-Mouse IgG- FITC | | 1 : 200 | Chemicon | AP308F |
| Goat Anti-Mouse IgM –FITC | | 1 : 200 | Sigma-Aldrich | F-9259 |
| Rabbit Anti-Mouse IgG- Texas Red | | 1 : 200 | Jackson | 315-075-003 |
| Goat Anti-Rabbit IgG–FITC | | 1 : 200 | Sigma-Aldrich | F-1262 |
| Goat Anti-Rabbit IgG–Texas Red | | 1 : 200 | jackson | 111075-003 |
| Goat Anti-Rat IgG–FITC | | 1 : 200 | Sigma-Aldrich | F-6258 |
| Rabbit Anti-Goat IgG–FITC | | 1 : 200 | Sigma-Aldrich | F-7367 |
